# The lifestylisation of healthcare? ‘Consumer genomics’ and mobile health as technologies for healthy lifestyle

**DOI:** 10.1016/j.atg.2015.02.001

**Published:** 2015-02-07

**Authors:** Federica Lucivero, Barbara Prainsack

**Affiliations:** Social Science Health and Medicine Department, King's College London, United Kingdom

**Keywords:** DTC tests, Beyond-the-clinic (BTC) genomics, m-Health, Symbolic order, Normative categories, Ethics

## Abstract

Consumer genomics and mobile health provide health-related information to individuals and offer advice for lifestyle change. These ‘technologies for healthy lifestyle’ occupy an ambiguous space between the highly regulated medical domain and the less regulated consumer market. We argue that this ambiguity challenges implicit distinctions between what is medical and what is related to personal lifestyle choices within current regulatory systems. In this article, we discuss how consumer genomics and mobile health devices give rise to new ways of creating (and making sense of) health-related knowledge. We also address some of the implications of harnessing, rather than denying, the hybridity of mobile health devices, being situated between medical devices and consumer products, between health and lifestyle.

## 1 Introduction

While genomics has traditionally been associated with reductionist approaches to health and disease, other newer 'omics symbolise interconnectedness and complexity (Meloni and Testa, 2014, Prainsack et al., 2014). Epigenomics in particular, by exploring how environmental stimuli ‘mark’ and alter the regulation of genes, emphasises the importance of behavioural factors for health. This resonates with the thrust of public health campaigns, for which behaviour change is an important way to increase health. National and international organisations devoted to the promotion of public health have highlighted the importance of behavioural change to improve wellbeing and prevention of disease (WHO, 2002, World Health Organization (WHO), 2008, The Evidence of Health Promotion Effectiveness, 2002, Department of Health, 2004).^1^ Central assumptions in these programmes are that people have the power to choose healthy or unhealthy lifestyles, and that they are thus at least partly accountable for their health (Buyx and Prainsack, 2012).

Perhaps unsurprisingly in this context, healthy living, or healthy *lifestyle*,^2^ has become central to the commercialisation of consumer products as well. As Sarah Nettleton put it, “lifestyle is a concept which has come to refer to people's styles of living, which, in turn, are shaped by their patters of consumption” (Nettleton, 2013).^3^ The commercialisation of consumer goods with remedial qualities^4^ has been seen to symbolise the rise of a new *petite bourgeois* culture of healthy lifestyles, in which people are seen as consumers (Featherstone, 1991). There is no area of research, it seems, that is not used for commercialisation of ‘personalised’ services to consumers: companies offer personalised health and diet recommendations on the basis of the microorganisms inhabiting their bodies,^5^ on their blood type,^6^ or on their DNA.^7^

Genomics has been a particularly active playground for personalised services marketed directly to consumers. For example, in the years 2000–2010 a plethora of companies offering so-called direct-to-consumer (DTC) genetic testing entered the market, providing information about genetic predisposition to diseases and traits (Prainsack et al., 2008, Kalokairinou et al., 2014). Many of them also offered advice on lifestyle changes. As pointed out by Saukko et al. (2010) for the case of nutrigenetic testing, *lifestyle* products have emerged as an alternative regulatory category to *medical* genetic tests. According to the authors, the label of ‘lifestyle products’ has been advanced by scientists who, while legitimizing the ‘seriousness’ of these tests, negotiated the space for a “hybrid or compromise category” that would stand “between medicine and consumer culture” (Saukko et al., 2010:751).

A renegotiation of the boundaries between medical and lifestyle products can be seen also in other areas. Digital mobile devices increasingly leave the gadget world to enter the medical domain. These devices include wearable sensors for the tracking of movements or physiological functions, mobile applications (‘apps’) for the calculation and analysis of caloric intake, or for monitoring sleep patterns and offering personalised advice. These products are marketed as tools to enable users to eat healthier, move more and become aware of ‘sustainable’ lifestyles. Initially appeared on the market as consumer products, these devices are increasingly being co-opted into the medical domain. Policy makers regard these products as having “the potential to play a part in the transformation of healthcare and increase its quality and efficiency” (EC, 2014: 3) while insurance companies consider scenarios wherein these devices can be used to monitor their customers' lifestyle to ultimately adapt their premium.^8^ These innovations occupy the ambiguous space between the highly regulated medical domain and the less regulated consumer market, where pre-market approval is easier to obtain and integration in the clinical pathway through public procurement is not required.

This ambiguous status of m-health devices and applications challenges the intuitive distinction between what is medical and what is instead related to personal lifestyle choices. In the following section we will show how regulatory questions raised by what we call ‘technologies for healthy lifestyle’, such as DTC genomics and m-health, signify a blurring of institutionally established normative categories. We will then reflect on how m-health devices and apps change the meanings of health information and propose new ways of creating (and making sense of) knowledge. Finally, we will address questions related to the blurring of the distinction of lifestyle v. medicine that are helpful for policy making.

## 2 Regulatory challenges and controversies

Technologies challenge established social values and meanings. Take the example of brain-machine interfaces and how, by blurring the distinction between physical bodies, minds and machines they question our definition of ‘body’ and ‘person’ (Lucivero and Tamburrini, 2007). Swierstra and colleagues^9^ argue that new technologies destabilise concepts that serve as a guide to classify reality, and that they create new interpretations (Swierstra et al., 2009: p 276). By doing so, new technologies challenge our symbolic order, that is, the grid of concepts that are used in a certain society to order and categorise reality. Changing meanings in turn raise new normative questions. This has happened in connection with molecular medicine, for example, which presented us to the idea that it is possible to be sick at the molecular level without the patients' experience of symptoms and introduced the concept of ‘biomarkers’ (Boenink, 2009). The latter shift challenges definitions of ‘healthy individuals’ vs. ‘patients’ (as subjects suffering from a symptom or disease) and requires healthcare systems to adapt to this new framework.^10^ As the concepts of ‘healthy’ and ‘sick’, also the labels of ‘medical’ vs. ‘lifestyle-related’ can be considered a dichotomy that seems to be assumed in European and North American regulatory tools. The unfolding regulatory debate around DTC genetic testing and m-health, described below, shows that new technologies for health and wellbeing present a hybrid character that destabilises some normative categories referring to the medical v. lifestyle-related distinction.

### 2.1 Direct-to-consumer genetic testing

In autumn 2007, two companies started offering online tests which would soon become a concern of health authorities and policy makers: *23andMe* in Mountain View, CA, and the Icelandic company *deCODE Genetics*, offered individual genetic risk calculations for fees starting at a few hundred dollars. Customers could purchase a ‘spit kit’ directly from the company, post it and, only few weeks later, access their genetic risk scores for a wide range of diseases, drug metabolism, and other characteristics. Other companies soon followed suit; a few weeks after *23andMe* and *deCODE Genetics*, *Navigenics* (Foster city, CA) started offering a similar service; and in 2009, San Diego-based *Pathway Genomics* became the fourth Personal Genomics (PG) company to offer SNP-based^11^ genome-wide risk predictions to consumers online. With the exception of *Navigenics*, which restricted the scope of their tests to important health conditions from the beginning, these companies offered ‘personalised’ risk calculations for a wide range of phenotypes and traits (e.g. diabetes, alcohol flush syndrome, eye colour), as well as results of SNP-based analysis of carrier status and drug response.^12^

Only a few months after these online services were set up, health authorities stepped in. During spring and summer 2008, the Department of Health of the state of New York and the California Department of Public Health sent letters to *23andMe* and *Navigenics* warning them of continuing to offer their services over the Internet without a genetic testing licence. Companies insisted that their legislation and regulation for clinical genetic testing should not apply to them, as their services did not intend to give medical information, but that they merely sought to educate and entertain their customers (see Prainsack, 2011). At the same time, however, these companies also made sure that they complied with relevant legal provisions — which meant that licensed physicians had to ‘order’^13^ the PG test, and DNA analysis had to be carried out in especially accredited laboratories. In the US, conflicts with regulators have since then continued, and reached a new peak at the end of 2013, when the US Food and Drug Administration (FDA) ordered 23andMe to stop offering health-related genetic risk information to *new* customers (Herper, 2013). The company complied with this, but kept health-related information visible to *existing* customers, and also accepted new clients to whom they disclosed only information on genetic ancestry which were not within the remit of the FDA. Although *23andMe* ceased to show the link between the SNP data and health risk information, almost overnight, a number of apps and services emerged that analysed people's SNP data — that the company enabled their customers to download – for health-related genetic probabilities. *23andMe* resumed the disclosure of health-related risk calculations from the UK in autumn 2014 (Gibbs, 2014).

In Europe, where PG testing is far less well known than in the US, genetic tests for medical use must be ordered, and results received by, a licensed physician. This is the case in Austria and Germany, for example; a difficulty regarding the interpretation of these laws lies in delineating what tests are medical and which ones are not medical. Some tests are clearly medical, e.g. when they look for a genetic variant that robustly correlates with a disease; however such robust correlations do not exist with many of the tests that companies such as *23andMe* perform. Similarly, within the EU, genetic tests are considered in vitro diagnostic medical devices if they are health-related (Directive 98/79 European Parliament and Council, 1998; see also Kalokairinou et al., in press). According to ongoing discussions on a draft regulation on medical devices,^14^ there is a need to intensify scrutiny of these tests, mandate the inclusion of healthcare professionals in the testing process, and include a wider range within the remit of the Regulation. The implications would be that any genetic test that provides ‘information with direct or indirect impact on health’ would be included in such Regulation (European Commission, 2012, European Parliament, 2013; see Kalokairinou et al., in press). This proposed framework does not seem to entirely solve the controversy. In fact, the existing ambiguity regarding ‘health-related tests’ would remain, as *any* genetic test can provide information with at least indirect impact on health if we interpret ‘impact’ widely.

### 2.2 Mobile health

According to a recent study, 97,000 mobile health apps are currently available, and it is predicted that the market will reach a value of $26 billion in 2017 (Research2guidance, 2013). In April 2014, the European Commission (EC) issued a Green Paper on mobile health. This document was accompanied by a staff-working document describing the “existing EU legal framework applicable to lifestyle and wellbeing apps”. In this document, the definition of m-health draws on a 2011 report of the World Health Organization, according to which m-health as component of electronic health (eHealth) consisting of “medical and public health practice supported by mobile devices, such as mobile phones, patient monitoring devices, personal digital assistants (PDAs) and other wireless devices” (WHO, 2011: 6). Building on this definition the EC Green Paper specified that mobile health “also includes applications (hereafter “apps”) such as lifestyle and wellbeing apps” (EC, 2014: 3)^15^ that is “apps intended to directly or indirectly maintain or improve healthy behaviours, quality of life and wellbeing of individuals” (*ibidem*). This definition includes medication reminders for chronic patients, apps offering fitness or dietary recommendation and sensors “collecting physiological, lifestyle, daily activity and environmental data” (*ibidem*).

In the Green Paper and related working staff document several regulatory and ethical challenges are addressed: the right to privacy and data protection, the applicable EU legal framework for quality control and certification, patient safety, applicable reimbursement models, liability issues, interoperability, possibilities for international cooperation and market potential, privacy, trust, disappointment and equal access. Some of these challenges seem to be strictly connected to the double nature of m-health as systems supporting both health practices and lifestyle/wellbeing-related behaviours. This emerges specifically in the section of the Green Paper that discusses the applicable EU legal framework to ensure the development and safe adoption of these products. As explained in the document, in the European Union, “there are no binding rules as to the delimitation between lifestyle and wellbeing apps and a medical device or in vitro diagnostic medical device” (EC, 2014: 11).

The aforementioned Medical Devices Directive (MEDDEV)^16^ and the in vitro diagnostic medical devices Directive,^17^ both currently under revision to become Regulations,^18^ suggest that the “intended purpose” is a criterion to establish whether products fall under the definition of a (in vitro diagnostic) medical device and consequently establish whether the MEDDEV framework applies. When MEDDEV does not apply, it is unclear what rules wellbeing and lifestyle apps have to comply with. To complicate things, as discussed above, during the drafting of the MEDDEV Regulation, the possibility of including the principle of “indirect medical purpose” to the definition of medical devices has been debated. This principle would prevent to leave the decision of the purpose to the subjective intention of the manufacturer and would expand the scope of the medical device regulation (Vollebregt 2014).

In the U.S., the FDA published some guidelines (2013) on how they intend to approach the regulation of mobile medical applications. According to this early guidance, the FDA intends to use a risk-based approach and only regulate apps that “are intended to be used as an accessory to a regulated medical device, or transform a mobile platform into a regulated medical device”.^19^ Therefore, many health apps that are seen to present only minimum risk are not expected to be subject of future regulation. Specifically, “health and wellness records (e.g. diet and weight logs) for consumers are not being considered for regulation” (Barton, 2012: p 2) because they are not expected to cause harm to users.^20^ Within this regulatory uncertainty, some app developers have decided to have their system go though the clearance process for medical devices. WellDoc, for example, is a web-based mobile platform for management of diabetes that “has introduced the first mobile prescription therapy for adults with type 2 diabetes-BlueStar” in June 2013.^21^ This app, whose developers filed and received FDA clearance (Iyer, 2014), offers a piece of software that has to be prescribed by the health provider and it will eventually be reimbursed by insurance providers as pharmaceutical diabetes treatments.

The ambivalent status of m-health applications, being situated between medical devices and consumer products, raises challenges related to certification and quality control, reimbursement, liability, and data protection.^22^ Moreover, the place of these applications in mainstream healthcare pathways is unclear. Legislators face the dilemma of either treating these products as non-medical devices — with the associated risks for consumers' safety and the non-adoption of these solutions in mainstream healthcare — or regulating them as medical devices — with the risk of unsustainable costs for this growing market of small and medium enterprises.

We argue, however, that this dichotomy can be surpassed, and that important lessons can be learned from the case of consumer genomics. Both consumer genomics and m-health raise questions concerning their regulatory status. Both have in common that they do not neatly fit into existing categories that legal and regulatory systems operate with. On the one hand, they differ from medical devices in that their direct clinical utility is often low. This is the case because the specificity and sensitivity of the test is very low, because they test phenotypes that are not directly clinically relevant, or because the data are not validated. Another important difference between technologies for healthy lifestyle and medical devices resides in the low risk for users' safety attributed to the first in comparison with the latter. On the other hand, the fact that they can be used for healthcare intervention — e.g. by a woman consulting her physician with a fertility issue bringing a several years of menstrual cycle data on a handheld device, or by a person informing her doctor that she may be a non-responder to certain blood-thinners following a consumer genomics test — raises issues about quality control; if such data are used in a clinical context and they need to be accurate.^23^ Rather than force them into existing categories, regulation should acknowledge that these products are hybrids between consumer and medical products and embrace, instead of reject the nature of their normative ambiguity. In the following we make a first step towards harnessing such ambiguity by looking at the way these devices challenge our way to make sense of medical knowledge and health information.

## 3 Changing meanings of medical information and expertise

Social scientists have shown that health information is never intermediated by technologies in a simple, straightforward way, but always “mediated” (Latour, 2005) in complex and unpredictable ways: “produced, distributed, regulated and used” via people, technologies, places (Wathen et al., 2008: p.3). This implies that in order to understand how devices for health and wellbeing challenge a society's normative framework, it is important not only to look at regulatory uncertainties, but also at how information is represented and understood in practices of use.

### 3.1 DTC genomics

As one of us argued in the 2008, when several genome-wide online testing services had just entered the market at the same time, regulatory authorities were accustomed to assume that a medical test is a distinct entity governed by a clearly discernible set of experts: doctors and public-health authorities. This no longer holds true. Genomics blurs the boundaries that make such clear distinctions possible. A genome scan reveals information that is medical, genealogical and recreational. And those who scan and interpret the data are not distinct bodies of experts, but instead, novel configurations of geneticists, customers, ethicists, bioinformatics experts and new media executives (Prainsack et al., 2008: 34).

The blurring of the boundaries between health-related information and information about other factors, such as hair curl or alcohol flush syndrome, stems from the fact that our genomes inevitably contains information on other aspects than only our health. While clinical genetic testing had filtered out the answers to specific clinically relevant questions from the ‘noise’ of the genome, personal genome-wide testing online contains information on any markers that had ever been studied, health-related or not. Some companies offering genome-wide tests online — such as bio.logis (bio.logis.com) based in Frankfurt, DE — restrict their analyses to clinically useful and actionable information (Vayena and Prainsack, 2013); others, such as *23andMe*, disclose anything that can be learned from genomic information, pertaining to health, ancestry, and other traits such as intelligence or skin colour. People use and make sense of this information sometimes, alone, sometimes with friends and family members, or even with their physicians.^24^ Also the use of this information can thus be health-related or not, depending on the meaning and utility that users attribute to this information. This is one of the most impactful ways in which online genetic testing has challenged existing categories and boundaries: it moved a practice to which clinicians had been the gate keepers – namely genetic testing that provided health-relevant information — to situations where clinicians were involved only marginally, or not at all.^25^

### 3.2 m-Health

Similarly, m-health technologies challenge established concepts of what constitutes “health information” or medically relevant information. As explained above, m-health comprises a diverse range of devices from self-tracking devices (sensors and mobile apps) for healthy people who want to be aware of their eating, sleeping, exercising patterns as well as their vital signs (heartbeat, blood pressure, etc.) to tools for patients with chronic conditions (diabetes, COPD, asthma, etc.) to help them manage their health. These devices collect different types of bodily data and are integrated in different practices. In the case of apps for diabetes management, for example, diabetic patients can measure the quantity of glucose in their blood through a regular or Bluetooth glucometer that sends them to the phone, where a specific app records readings on a logbook, shows statistics and trends, calculates the insulin intake they have to consume offers personalised advice and educational tip based on the patient's input, and eventually shares information with the doctor.^26^ Similarly, in case of patients with asthma, commercially available apps offer self-assessment tools as peak expiratory flow rate, symptoms, triggers and medication diary whose information is translated in charts that can be shared with GPs to change treatment.^27^ In these cases, it is healthcare providers' experiences, patients' understanding of their conditions and the medical knowledge encoded in the app's software that renders the collected data actionable. In the case of ‘wellbeing apps’ that are targeted at people without specific conditions, the data detected by the app and the way it is processed, produces information that is not clearly related to health outcomes either. In the case of apps for measuring sleeping and exercising patterns, calories intake, or blood oxygen level, it is unclear how the extracted data about people's behaviour and lifestyle has a health impact and can be used for medical purposes as the correlation between this information and health conditions are still uncertain.

As in the case of online genetic testing, these types of self-tracking technologies disclose a broad range of information that is not clearly medically relevant and that involves clinical professionals very marginally if at all.^28^ Biomedical technologies, therefore, mediate our way of interpreting and understanding medical information in at least two ways. First, they expand the meaning of what is health relevant data, putting a diverse range of biological data into a health context (as it happens in the case of the Apple Inc. Health app, which integrates in the same dashboard health and fitness information from different apps and helps consumers ‘accurately’ answer the question ‘how are you?’, as the webpage recites^29^). Second, by directing their marketing efforts towards consumers, they challenge the traditional distinction between professionals as experts who manage a body of knowledge and make clinical decisions on the one hand, and patients, supposedly lacking such expertise, on the other.^30^ These changes in meaning challenge our current normative structure and raise normative and political questions concerning a broad set of issues: e.g. the roles and responsibilities of existing actors (e.g. health professionals, app developers and consumers), the need to create new professional intermediary figures, the accuracy and rational behind the interpretation of sensor-gathered data and the role of such information and tools in (non-)medical decision making.

## 4 Conclusions

The strive for lifestyle change as a means for better health outcomes has met the private interest of companies offering technological tools for this purpose. DTC genetic tests and mobile health applications are two examples of these ‘technologies for healthy lifestyle’. In this article we have discussed how these exemplary technologies challenge traditional distinctions that are entrenched in our normative and regulatory frameworks. For example, the distinction between clinical care and self-administrated wellbeing helps to set criteria for reimbursement policies as well as deciding which devices need special certification because they raise particular safety issues and which do not. Apps for wellbeing and lifestyle blur these boundaries and require a reconsideration of the distinctions underpinning these frameworks. Such a conceptual and normative clarification is important for a number of reasons related to regulatory challenges: 1) to establish what relevant regulation applies, in the context of quality certification or reimbursement policies; 2) to define roles and responsibilities in case of liability issues; 3) to define how these apps should enter — what the EC calls — “the main stream of healthcare provision” (EC, 2014: p. 12).

In this paper we have discussed how regulatory debates are unfolding in the cases of beyond-the-clinics genetic tests and m-health. We have highlighted how in both cases the regulatory challenges are associated with a hybrid character of both products that do not fall in the current definition of ‘medical device’, but are also too focused on medical practices and health domains to be considered as regular consumer products. This piece makes a plea for further analysis of such a normative ambiguity and finally offers few questions that need to be addressed in this direction.

A first set of questions that need to be addressed concerns the hybrid character of these products between the categories of consumer/lifestyle related and healthcare/medical devices. First of all, this hybridity needs to be unpacked: how are its multiple dimensions of meaning and value configured in specific practices and products? Also, its implications need to be explored: for example, does this hybridity require some system to ascertain the quality of products that do not clearly fall in the definition of medical device and yet have a strong impact on medical and healthcare practices? Some action has already been taken in the direction of offering an authoritative assessment of the multitude of available health apps: the European Directory of Health Apps^31^ released in October 2013 and compiled by Patient View (a UK-based research, publishing and consultancy group) as well as the App Library on NHS choice^32^, for example, provide guidance to people who navigate the existing market of health apps. They also offer advice on ‘safe and trusted apps’. If these types of assessments become more common and widespread — and we believe that they should — what kinds of bodies should carry them out? And what would be the criteria for such a body to evaluate apps as ‘safe and trusted’?

A second group of questions concerns the appropriateness of the notion of ‘intended use’ by the manufacturer as a criterion to assign a product the status of medical device. Should we abandon this criterion and discuss instead the ‘imagined purpose’ as defined by users? If the regulatory goal is to protect users from harm, then the benchmark must be the actual use of a tool or technology, and not how the manufacturer or provider intends it to be used. At the same time, looking at actual use entails significant challenges regarding its operationalisation. Especially for market approval, how can actual use be assessed before a tool or device has been released? Would representative samples of ‘test users’ be required whose actual use could determine the category under which a new tool or device would be subsumed? In this way different rules would apply if I say that my purpose is to know more about my ancestors or to know more about my health. Furthermore, the portable character of mobile health devices, i.e. the fact that they can be easily and discretely carried around by individuals, makes the physical and social context relevant for the definition of what is medical and what is not. When these health devices move ‘beyond the clinic’, how to take into account the always varying social factors that make the collected data and suggested action health (or medically) relevant or not?

Finally, it is often unclear who the experts for the interpretation of particular health-relevant data are. For example, for online genetic tests, general practitioners — who often have not been trained extensively in genetics — may not be the best experts to entrust with this. Who will fill this ‘interpretive vacuum’ (see also Beckmann, 2014)? Authors have discussed the trends towards personalised medicine and the (bio)medicalisation of everyday life (e.g. Clarke et al., 2010). However, new technologies and practices provide a wealth of data on individuals at various stages of health and disease that go beyond people's biological make up but include information also on people's interests, hobbies, priorities and lifestyle. Should we start to explore the trend towards “lifestylisation” of healthcare,^33^ in which treatments are not only personalised to the person's genetic makeup but also to the individual's lifestyle?

## Footnotes

1 See also the amount of informative material on how to change eating and fitness habits, offered by the Weight control Information Network (WIN) an information centre of the National Institute for Diabetes and Digestive and Kidney Diseases at the US Department of Health and Human Services: http://www.win.niddk.nih.gov/publications/ [Accessed on 6th January 2015].

2 For a sociological perspective on concept(s) of lifestyle, philosophies of wellbeing and health promotion schemes, see O'Brien (1995). In this essay, the author shows how the concept of lifestyle, initially referred to individual choices, has increasingly been used as a “vehicle for differentiating a population” (193) in a consumerist and market oriented culture. The association between health and lifestyle has, according to O'Brian, been a political construction together with the emergent role of the concept of “wellbeing” in health promotion strategies.

3 The Oxford English Dictionary (OED) defines “lifestyle” as a “style or way of living (associated with an individual person, a society, etc.); *esp*. the characteristic manner in which a person lives (or chooses to live) his or her life.” The OED definition of compounds such as “lifestyle advice”, “lifestyle change”, “lifestyle factor”, etc., recites “Of or relating to the way in which one lives (or chooses to live) one's life, esp. with regard to quality of life”. http://www.oed.com/view/Entry/108129?redirectedFrom=lifestyle#eid [Accessed on December 1st, 2014].

4 See Tomes (2001) for a history of consumer culture and its relationship with medicine in the period 1900–1940.

5 http://ubiome.com/#how-it-works (Accessed on January 6th 2015).

6 http://www.dadamo.com/.

7 http://mydietclinic.com/services/nutrigenomix-testing/ (Accessed on January 6th 2015).

8 Such insurance policies are currently explored in Europe by the Generali Group that, within the next 12 to 18 months, plans to offer policies that reward healthy people, based on the information provided by their tracking devices (http://www.sueddeutsche.de/news/wirtschaft/versicherungen-versicherer-generali-will-fitnessdaten-von-kunden-sammeln-dpa.urn-newsml-dpa-com-20090101-141121-99-02990 [Accessed on January 6, 2015]).

9 In their article, the authors build on the concept of ‘symbolic order’ elaborated by anthropologist Mary Douglas (Douglas, 1966) and on the idea of technologies as ‘monsters’ discussed in Smits, 2006.

10 Similarly, the concept of ‘patients in waiting’ proposed by Timmermans and Buchbinder (2010) captures the liminality of patients involved in screening trajectories that place them in a category in between normal health and pathology.

11 Single nucleotide polymorphisms (SNP) are variations in the DNA at the level of single bases (nucleotides: A, T, C, and G).

12 While people celebrated this development as a new era of patient empowerment and the democraticisation of medicine, others were concerned about the questionable robustness of the scientific evidence underpinning personalised risk calculations (e.g. Janssens et al., 2008), or about the fact that these companies cut out medical professionals; in the early days of personal genomics tests online, companies operated according to the ‘pure’ direct-to-consumer model (Prainsack and Vayena, 2013), and commentators were concerned that lay people would not be able to understand the probabilistic information given to them by the companies (e.g. Hunter et al., 2008).

13 In practice the physicians only needed to sign off the order, without ever having met with the test-taking person; see also Dvoskin and Kaufman, 2011.

14 See the reference to the MEDDEV draft regulation below.

15 The WHO report is based on a survey conducted by the Global Observatory for eHealth in 2009 and completed in 119 member states. This survey contained a section dedicated to m-health and including fourteen categories of m-health services such as: health call centres, emergency toll-free telephone services, managing emergencies and disasters, mobile telemedicine, appointment reminders, community mobilization and health promotion, treatment compliance, mobile patient records, information access, patient monitoring, health surveys and data collection, surveillance, health awareness raising, and decision support systems (WHO, 2011: 12). It is a sign of the times (the survey is from 2009) that “apps” and Internet-based services on smart phones are not explicitly referred to in the report.

16 Directive 93/42/EEC on medical devices.

17 Directive 98/79/EC on in vitro diagnostic medical devices.

18 In EU law, the difference between a directive and a regulation is that member states have more leeway in deciding how to implement a directive, while a regulation immediately becomes a nationally enforceable law (i.e. regulations are self-executing).

19 Source: http://www.fda.gov/MedicalDevices/ProductsandMedicalProcedures/ConnectedHealth/MobileMedicalApplications/ucm255978.htm#c [Accessed on January 6, 2015].

20 On January 20, 2015, after the submission of this article, the Centre for Devices and Radiological Health released a Draft Guidance for Industry and FDA staff clarifying the difference between “low risk general wellness products” and regulated medical devices: “CDRH defines general wellness products as products that meet the following two factors: (1) are intended for only general wellness use, as defined in this guidance, and (2) present a very low risk to users' safety. General wellness products may include exercise equipment, audio recordings, video games, software programs and other products that are commonly, though not exclusively, available from retail establishments (including online retailers and distributors that offer software to be directly downloaded), when consistent with the two factors above”. Source: http://www.fda.gov/downloads/MedicalDevices/DeviceRegulationandGuidance/GuidanceDocuments/UCM429674.pdf [Accessed on January 30, 2015].

21 Source: https://www.bluestardiabetes.com/ [Accessed on January 6, 2015].

22 The list of the legitimate grounds allowing processing of personal data is more restrictive in the case of health-related data.

23 Another example of the role of m-health in healthcare intervention is visible in the scenarios drawn by the developers of ginger.io (https://ginger.io/). This application automatically gathers and analyses data retrieved through mobile phones' sensors and prompts health providers to take action. At the moment it is tailored to detect behavioural changes of people with depression or other behavioural disorders. This tool translates movement data in health-related information which is expected to support healthcare providers to take action. Although it is not a medical device, the strong integration of such tool into the healthcare context implies that it cannot simply be considered as a consumer product, and a quality control is necessary.

24 Although it has been remarked also that healthcare professionals, like physicians and pharmacists, often lack the appropriate genetics education to interpret the test results and use the gained information to deliver better healthcare to their patients (Patrinos et al., 2013).

25 To acknowledge the different formats of clinicians' involvement in online genetic testing, which ranges from ordering the tests on behalf of their patients to hearing about it only when their patient discloses some results to them, Barbara Prainsack and Effy Vayena introduced the notion of beyond-the-clinic [BTC] genetics. See Prainsack and Vayena (2013).

26 Several apps for tracking and management for diabetes patients are available. See for example, https://www.bluestardiabetes.com/ and https://itunes.apple.com/us/app/ihealth-gluco-smart/id571576516?mt=8.

27 http://www.asthmamd.org [Accessed on December 2, 2014]. See also Huckvale et al., 2012 for a systematic review of asthma apps.

28 Interestingly, business models of health and wellbeing apps are very diverse at the moment and are directed not only towards individual consumers, but also GP, insurance companies, public services, employees (see also: http://medcitynews.com/2014/04/mobile-health-business-models-still-work-progress/ [Accessed on January 7, 2015]).

29 See https://www.apple.com/ios/whats-new/health/ [Accessed on January 7, 2015].

30 This distinction is currently challenged from different fronts. Besides the type of biomedical technological and scientific innovation discussed here, there are several types of service innovation in public health programmes that aims at giving a central role to patients. The Expert Patients Programme established by the NHS in UK in 2007 aims at offering self management courses to people living with chronic disorders http://www.nhs.uk/NHSEngland/AboutNHSservices/doctors/Pages/expert-patients-programme.aspx (Accessed on December 17, 2014).

31 Available at http://www.patient-view.com/-bull-directories.html.

32 Available at http://apps.nhs.uk/.

33 See also O'Brien (1995) who refers to “styled” health as an on-going trend since the 1980s in opposition to the medicalisation of everyday life and Paula Saukko contribution at the European Association for the Study of Science and Technology: “Contestation between evidence based medicine and lifestylisation of medicine: the case of online genetic testing” (EASST conference, Torun Poland, 17–19 September 2014).
